# Spatial heterogeneity of *Pelagia noctiluca* ephyrae linked to water masses in the Western Mediterranean

**DOI:** 10.1371/journal.pone.0249756

**Published:** 2021-04-07

**Authors:** Marina Pastor-Prieto, Nixon Bahamon, Ana Sabatés, Antonio Canepa, Josep-Maria Gili, Marta Carreton, Joan B. Company

**Affiliations:** 1 Institut de Ciències del Mar (ICM-CSIC), Barcelona, Spain; 2 Escuela Politécnica Superior, Universidad de Burgos, Burgos, Spain; Evergreen State College, UNITED STATES

## Abstract

*Pelagia noctiluca* is the most common jellyfish in the Western Mediterranean Sea, living in oceanic waters with a holoplanktonic lifecycle. Frequent outbreaks have been well documented in coastal areas, yet little is known about their offshore distribution. In this study we address the relationship between oceanographic structures and the distribution of *P*. *noctiluca* ephyrae along the central continental slope of the Western Mediterranean, covering a wide latitudinal gradient, during July-August 2016. The region is characterized by a rich and complex mesoscale surface circulation driven by the inflow of Atlantic Water into the Western Mediterranean through the Strait of Gibraltar. The results revealed a high variability in the ephyrae spatial paterns related with different water masses and the resulting mesoscale hydrographic features. Their horizontal distribution showed a clear latitudinal gradient with high abundances in the south, associated with recent Atlantic Water, and low abundances or absence in the north, in coincidence with the old Atlantic Water transported by the Northern Current. Ephyrae showed diel vertical migrations of short-extent in the first 50 m, with a wide distribution above the thermocline and the Deep Chlorophyll Maximum during daytime, being more concentrated towards the surface at night. The results suggest the population connectivity of *P*. *noctiluca* between the Atlantic and the Mediterranean. In that case, the abundance variability of the species in the Mediterranean could be modulated by its entrance associated with the inflow of Atlantic Water through the Strait of Gibraltar.

## Introduction

Jellyfish are conspicuous components of pelagic communities that show increases in population size often resulting in mass occurrences, or blooms, worldwide [1,2]. While there is a lack of scientific consensus in identifying global trends in jellyfish blooms [3], their negative impacts on human activities in coastal waters are remarkably increasing in frequency and severity [4,5]. These increases in jellyfish abundance have the potential to alter the balance of trophic pathways between smaller zooplankton and their predators in marine ecosystems [6]. Jellyfish distribution and aggregation are determined by the combination of environmental conditions and life history events resulting in a rapid increase in population numbers [7]. Hydrodynamic structures such as currents, fronts and eddies may act as mechanisms for their transport or confinement [8,9] thereby contributing to an increase in mesoscale spatial heterogeneity. These structures support high levels of biological activity e.g. [10,11] controlling the interactions among organisms with limited horizontal mobility [12] such as jellyfish. Differently, they have the ability to actively swim vertically through sharp clines [13].

The Western Mediterranean Sea (WM) is characterized by a complex physical dynamics with distinctive traits, especially in regard to the thermohaline circulation. The surface circulation is mainly driven by the inflow of Atlantic Water (AW) through the Strait of Gibraltar, its signature being modified as it travels eastward [14]. The input flow of AW in the WM follows the north African coast creating anticyclonic eddies [14,15] which can be trapped by the bottom topography of the Alboran Sea or freely displaced around the Algerian basin and reach the Balearic Islands. The Balearic Islands can be considered a transitional region between the two main WM sub-basins: the Liguro-Provençal and the Algerian basins. Part of the AW flows across the Balearic channels forming the Balearic Current [16,17] that follows the northern side of the Balearic Islands to the west coast of Corsica [18,19]. A surface front, which is not deeper than 200 m, associated with the Balearic Current, separates recent AW brought by the current from the resident waters of the centre of the northern part of the basin [20]. Typically, the salinity of recent AW is nearly 1 unit lower than the older resident AW waters [21]. On the eastern side of the Liguro-Provençal basin, the recent AW flow from the Balearic basin joins the old AW from the Tyrrhenian Sea [22], forming the Northern Current which flows southwestwards along the continental slope, adapted to the bathymetry and contouring the northwestern basin cyclonically [23,24].

*Pelagia noctiluca* is the most common jellyfish in the Mediterranean Sea, living in oceanic waters [25,26]. It is a holoplanktonic species with a variable reproductive period depending on the region, and the presence of ephyrae has been reported throughout the year [27–29]. In the WM, the highest abundance of *P*. *noctiluca* occurs during spring and summer [28,30,31] and their blooms appear to be increasing in frequency and duration [26,32,33]. The studies on the spatial distribution of *P*. *noctiluca* in relation to hydrodynamic structures in open sea waters of the basin are scarce and restricted to some areas of the northwestern region. These studies have shown that the species is particularly abundant in the vicinity of the shelf-slope front associated with the Northern Current [28,34,35]. The particular hydrodynamic conditions of that region enhance and maintain high levels of biological production [36–38] providing ideal conditions for feeding, growth and reproduction of the zooplanktonic organisms. However, the lack of knowledge on the distribution of *P*. *noctiluca* in open waters further south limits our understanding of a large-scale picture of its spreading patterns in the WM.

Jellyfish are difficult to sample quantitatively [39,40] and the mechanisms of jellyfish transport and aggregation cannot be understood without a large scale sampling of their abundances combined with synoptic environmental measurements. In contrast, ephyrae can be efficiently collected with plankton nets and its distribution can be a good proxy of the global abundance and distribution of the species [35]. Considering this approach, the objective of the present study was to identify how the mesoscale water dynamics shapes the spatial structure of *P*. *noctiluca* ephyrae along the continental slope of the WM. To this aim, we performed an extensive plankton and hydrographic sampling, with a wide latitudinal and vertical coverage, that will result in a general view of the distribution of the species driven by hydrodynamic processes in open waters of the WM, and provide new insights on its potential populations increase.

## Material and methods

### Field sampling

**Fig 1:**
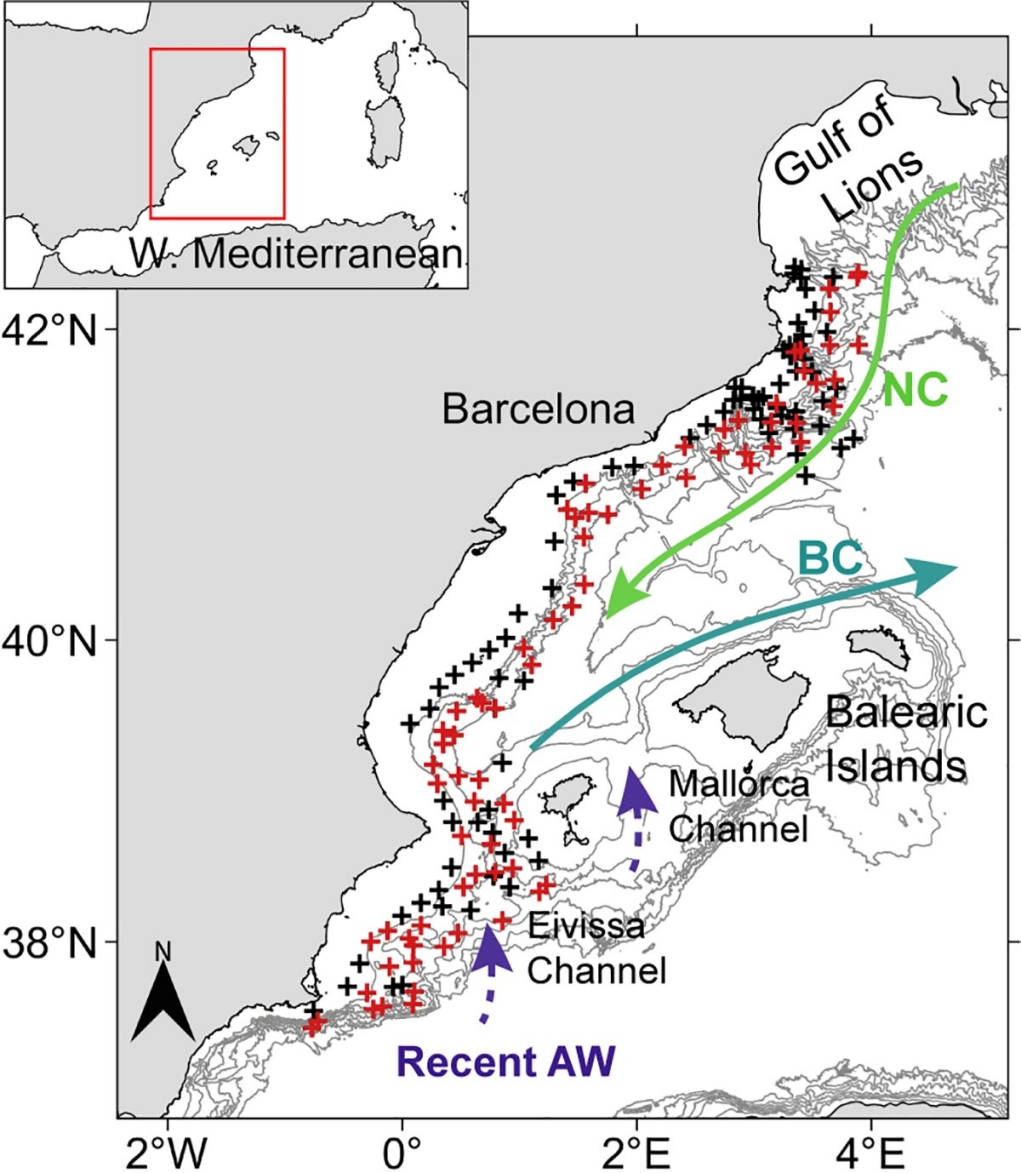
Study area in the Western Mediterranean. Hydrographic (black and red crosses) and plankton (red crosses) stations during the survey. The schematic lines indicate the main currents, Northern Current (NC) and Balearic Current (BC), and recent AW inflow through the Eivissa and Mallorca Channels. Coastline is from Natural Earth [41] and Instituto Hidrográfico de la Marina [42], and isobaths (every 400 m depth) are from European Marine Observation and Data Network (EMODnet, [43]).

The study was conducted on the central continental slope of the WM along a wide latitudinal gradient (37.4°N—42.3°N) in a south north direction, between 22^nd^ July and 28^th^ August 2016 on board the R/V García del Cid (Fig 1). A total of 170 hydrographic stations, with plankton sampling at 75 stations (29 at night and 46 during the day) were completed. At each hydrographic station, vertical profiles of basic hydrographic variables (salinity, temperature and fluorescence), from surface to 200 m depth, were obtained by means of conductivity-temperature-depth profilers (CTD) (SBE25 and SBE911), equipped with a fluorometer. At the plankton stations, hydrographic parameters were measured with a CTD (SBE3F) integrated in the plankton net. CTD data were inter-calibrated to make them readily comparable. Afterwards, data were bin averaged at 1 m depth intervals.

Zooplankton was sampled with a Multiple Opening/Closing Net Environmental Sensing System (MOCNESS net) of 1 m^2^ opening mouth and a 300 μm mesh. Hauls were oblique from deep to shallow waters, at a vessel speed of 1.2 ± 0.4 knots. The depth strata examined were: 200–100, 100–50, 50–25, 25–0 m, and the volume of filtered water was recorded by a flowmeter attached to the mouth of the net. Immediately after collection, zooplankton samples were fixed in 5% formaldehyde buffered with sodium tetraborate.

### Data analyses and sample processing

2D maps showing the latitudinal variation of surface salinity and temperature data were produced by applying Data-Interpolating Variational Analysis (DIVA) gridding to 10 m depth CTD data, using the Ocean Data View (ODV) software [44]. DIVA gridding was also applied using ODV, for creating sections of the depth variation with latitude of the upper 100 m depth from environmental data collected at all the stations. In addition, daily salinity and temperature at 10 m depth reanalysis products from Copernicus Marine Environment Monitoring Service (CMEMS, [45]) were used to estimate mean salinity and temperature values for the surrounding areas during the study period, and were represented with QGIS v3.4.11 [46]. Mean currents data for August 2016, at 10 m depth, from CMEMS were represented through QGIS v2.18.28 [47].

In the laboratory, the zooplankton samples were examined using a stereomicroscope in order to identify and count the ephyrae of *P*. *noctiluca* (total body diameter: 0.55–3.55 mm, S1 Fig). The number of individuals within each depth strata was standardized to number per 100 m^3^ of filtered water.

A preliminary exploration of the ephyrae vertical distribution, from surface to 200 m depth, was carried out for 12 of the 75 sampled stations, randomly selected and distributed along the sampling area. Considering these stations, 99.9% of ephyrae were found between 0–50 m depth. Based on these results, only 0–25 m and 25–50 m depth levels were considered for the vertical distribution analysis. For the mesoscale horizontal distribution, the two depth levels were grouped together (0–50 m). The effect of light (day/night) and depth on the ephyrae vertical distribution was analysed through a Generalized Linear Mixed Model (GLMM) (see S1 Equation in supporting information). A GLMM was also fitted to assess the effects of independent (Pearson’s cross-correlation coefficient < 0.5) oceanographic variables (mean surface, 5–10 m depth, salinity and temperature) from CTD data on the horizontal distribution of the ephyrae (see S2 Equation in supporting information). In order to avoid any spatial lack of independence among close sampling stations, the geographical position of each station was included as a random effect in both GLMMs. In addition, in both analyses the error family distribution used was a negative binomial, due to the patchy distribution of ephyrae (a normal condition in plankton ecology [48]), and with a log-link to avoid predicting negative numbers of ephyrae, using the “*glmer*.*nb*” function from the “MASS” package [49]. To reduce the bias due to different filtered volumes by the nets (mean 471 m^3^ ± 124 standard deviation) the (log-transformed) volume of filtered seawater was included as an offset inside GLMMs [50]. The GLMMs were carried out using the statistical programming language R v3.5.3 [51].

## Results

### Hydrographic conditions

**Fig 2:**
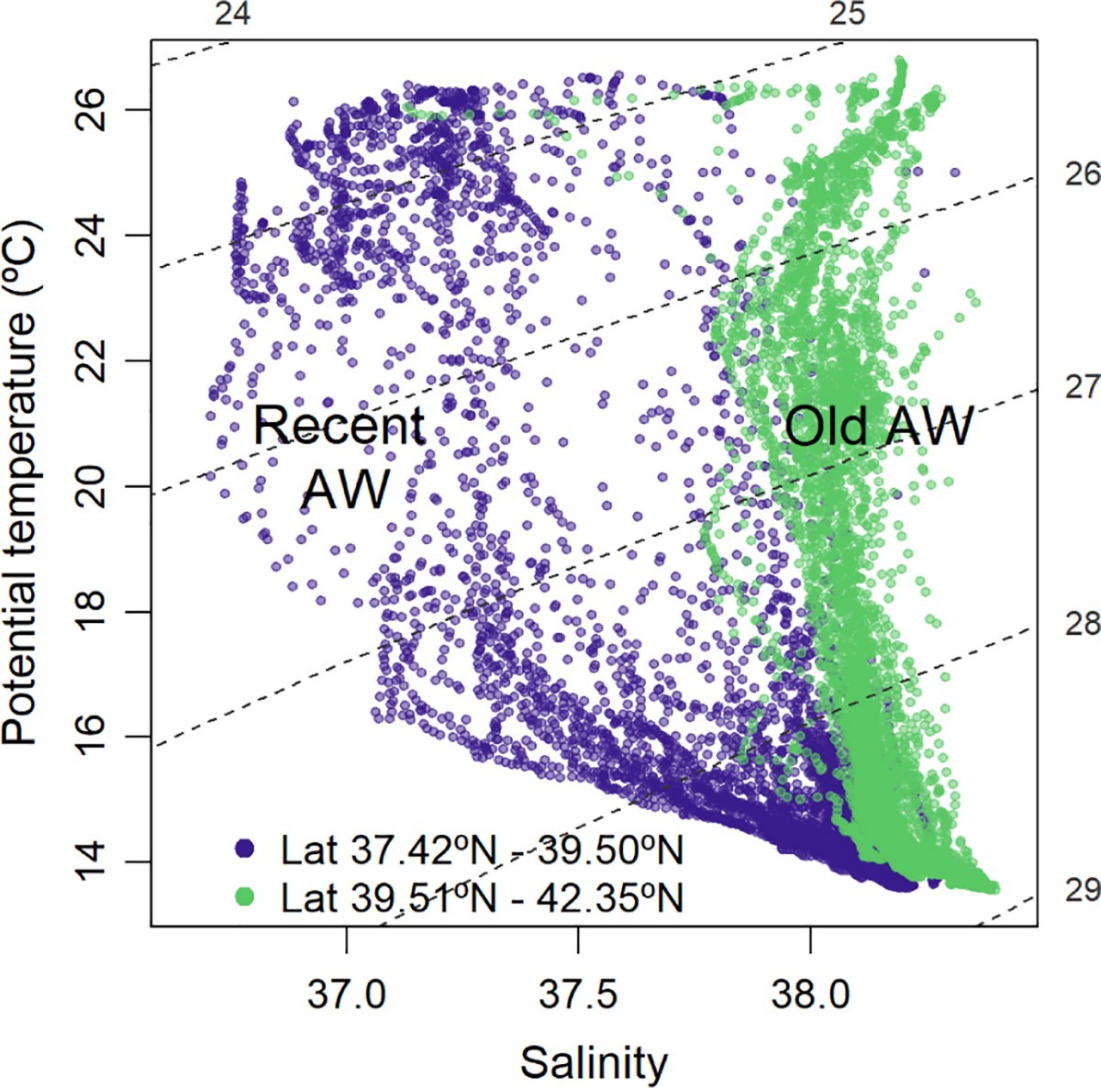
TS diagram of the water layer from 0 to 100 m depth. Recent Atlantic Water (blue dots), south of 39.5°N; old Atlantic Water (green dots), north of 39.5°N.

The TS diagram of the upper 100 m showed the more recent AW, characterized by relatively low salinity, and the old and more saline AW transported by the Northern Current, that stayed longer time in the basin (Fig 2).

**Fig 3:**
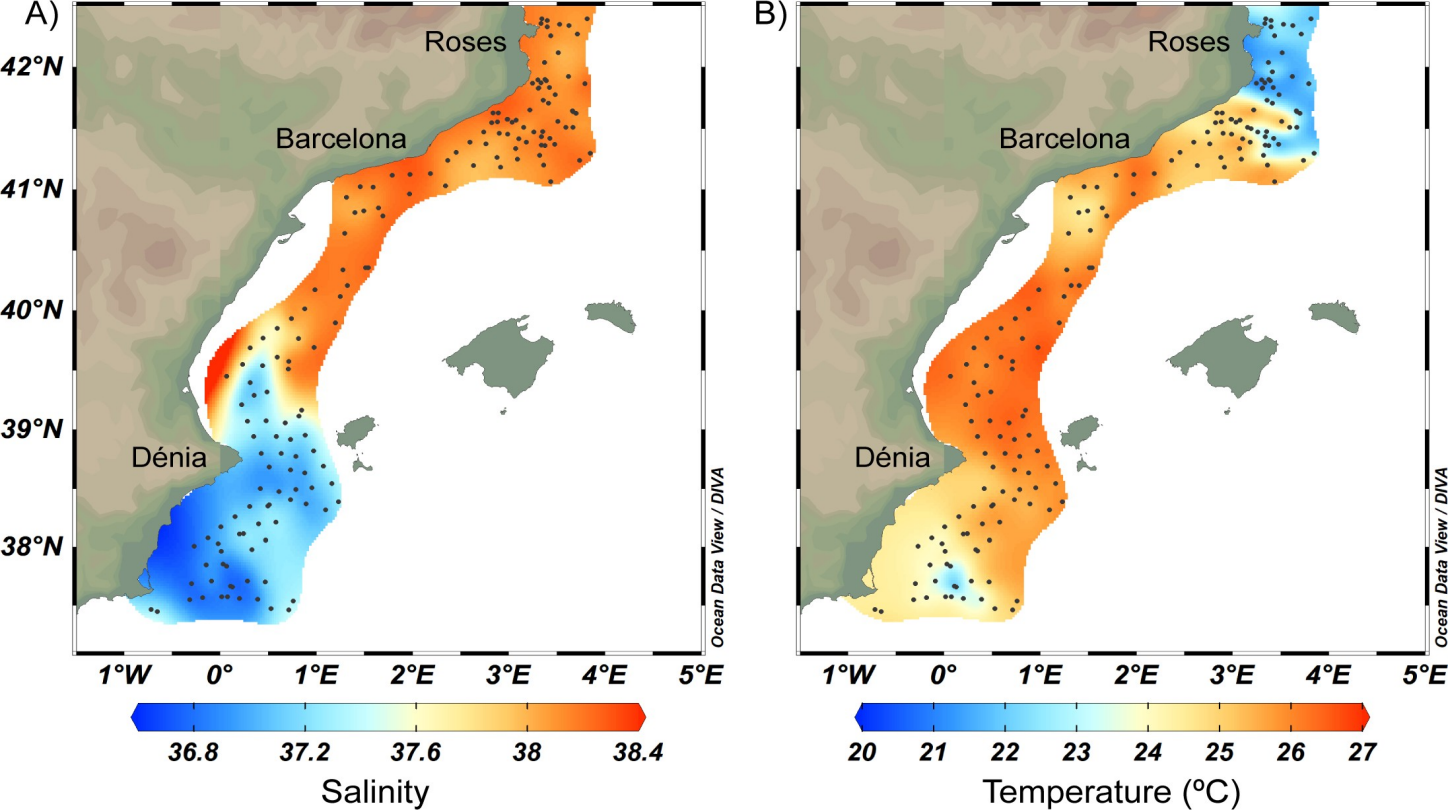
Horizontal distribution of a) salinity and b) temperature at 10 m depth from CTD data. Dots show the sampling stations. Images were created using ODV [44].

The spatial distribution of sea surface salinity (10 m) contrasted between the south (≈ 37.1) and the north (≈ 38.1) with a marked gradient between 39.5°N and 40.0°N, clearly separating the recent AW to the south and the old AW to the north (Fig 3A). Temperature (10 m) showed values around 24.0°C (23^rd^ July– 26^th^ July) in the southern part of the area, south of 38.3°N. Warm waters (≈ 25.2°C, 27^th^ July - 23^rd^ August) were detected in the central zone, while the northernmost part of the area presented the lowest temperatures (≈ 22.0°C, 24^th^ August– 28^th^ August) (Fig 3B).

**Fig 4:**
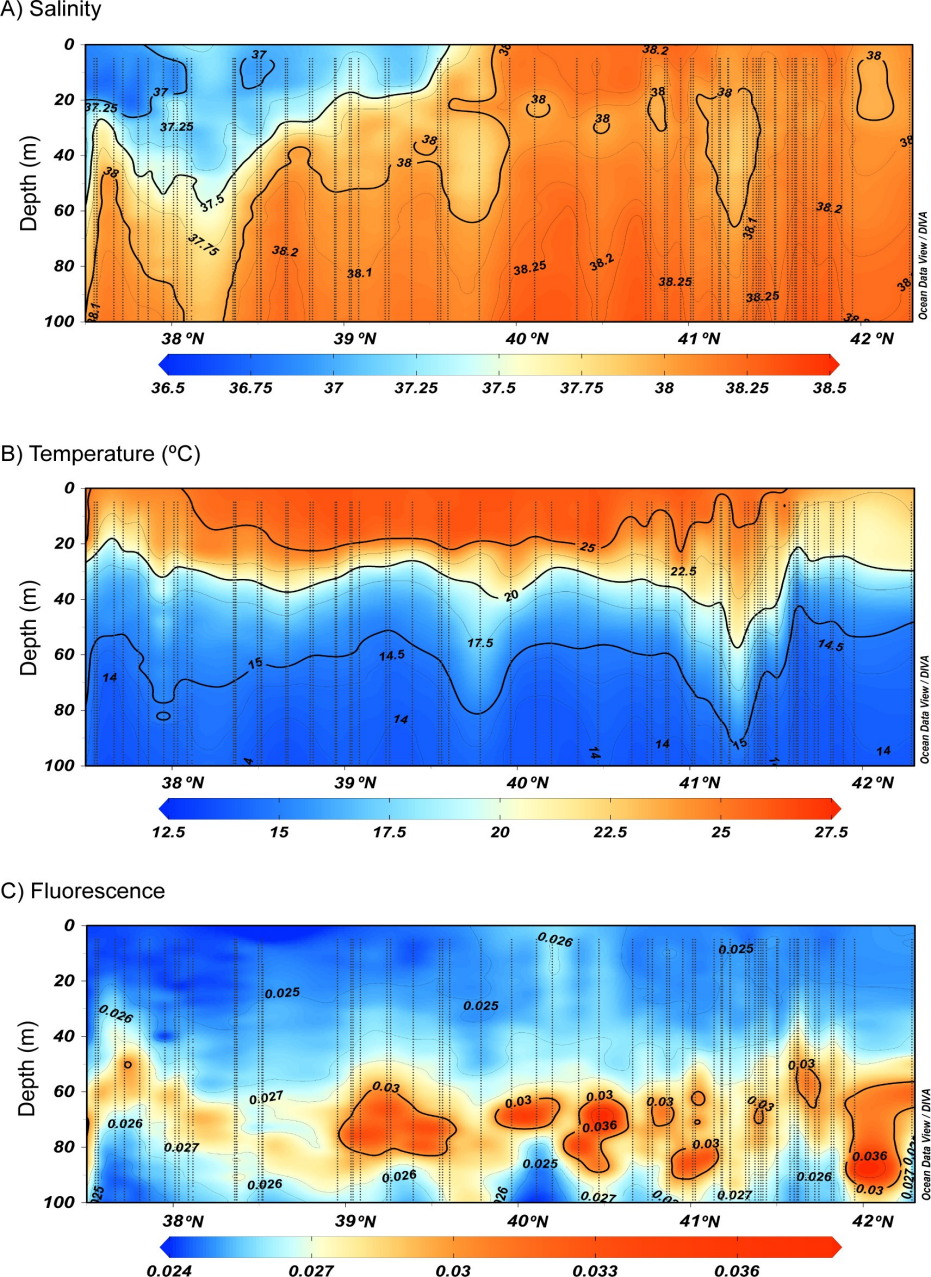
Vertical distribution of a) salinity, b) temperature and c) fluorescence in the upper 100 m depth from CTD data, along the continental slope. Horizontal axis indicates latitude range. Vertical dotted lines represent 1 m binned CTD profiles data.

The vertical section of salinity (Fig 4A) suggested the presence of a saline front between 39.5°N and 40.0°N, delimiting the presence of recent AW in the south and the old AW in the north. The recent AW was detected in the upper 60 m depth in the south and became shallower until 39.5°N (Fig 4A). The vertical section of temperature along the continental slope showed a surface mixed layer of about 20 m thickness, with temperature values above 25.0°C between 38.0°N and 41.0°N (Fig 4B). This central zone also showed the strongest thermocline gradient below the mixed layer, between 20 and 30 m depth. The thermocline becomes weaker south of 38.0°N and north of 41.0°N, with slightly lower surface temperature (Fig 4B). The vertical section of fluorescence was typical of the season with a clear Deep Chlorophyll Maximum (DCM) below the thermocline, between 50 and 90 m (Fig 4C).

**Fig 5:**
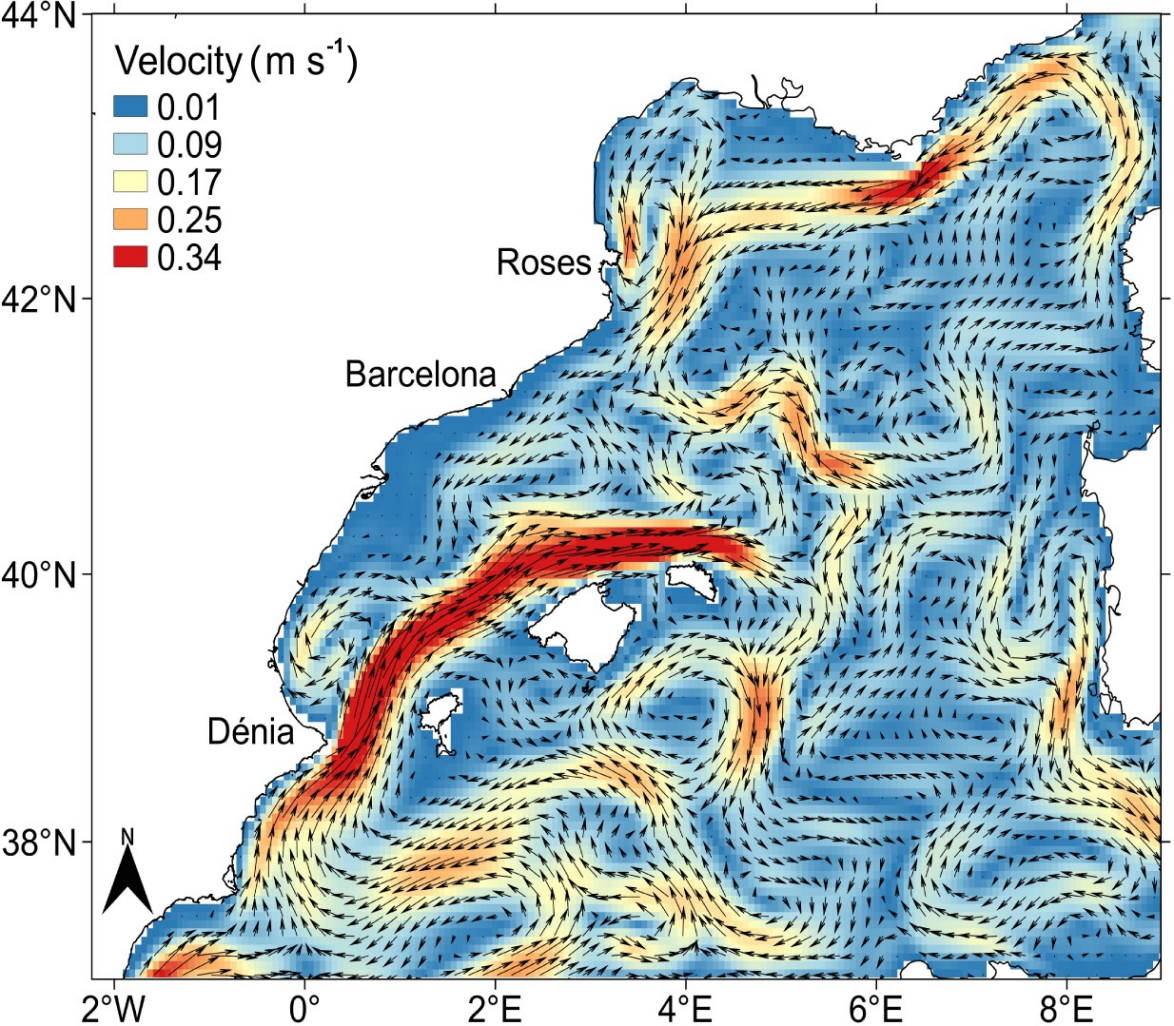
Mean velocity field for August 2016 at 10 m depth from CMEMS reanalysis [45]. The current velocity is depicted through the colour scale and arrows show the current direction. Coastline is from Natural Earth [41] and Instituto Hidrográfico de la Marina [42].

The surface currents were characterised by the strong jet of the Balearic Current flowing to the northeast along the northern coast of the Balearic Islands (Fig 5). This current displayed a deflection towards the northwest, at around 4.0°E, joining the Northern Current path. The presence of the Northern Current was evident in the northern part of the area flowing to the southwest along the continental slope (Fig 5).

### Vertical distribution of *P*. *noctiluca* ephyrae

**Fig 6:**
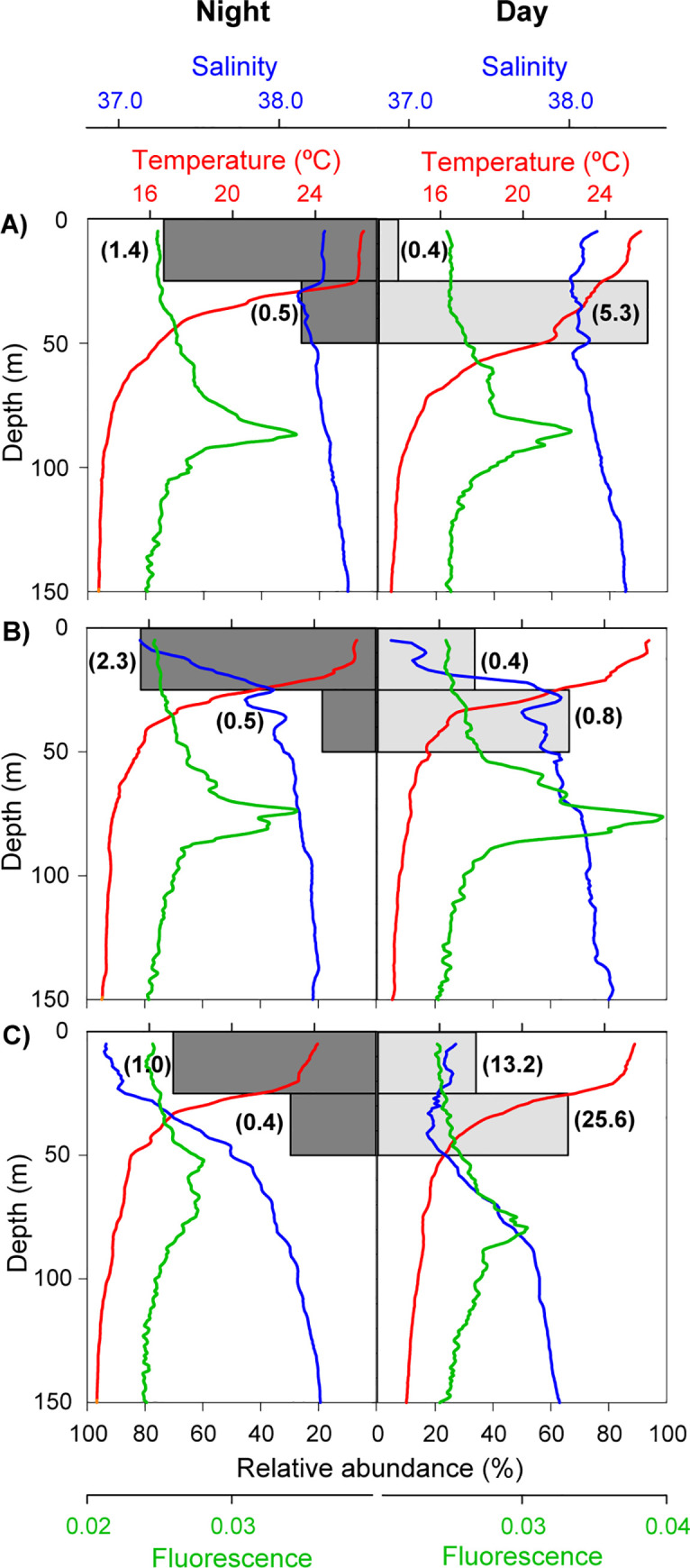
**Vertical distributions of ephyrae in night time (dark grey bars) and daytime (light grey bars) and temperature (red), salinity (blue), and fluorescence (green).** Data of six stations representative of the hydrographic conditions at the a) north (41.0°N, 2.0°E), b) centre (39.5°N, 0.5°E) and c) south (38.0°N, 0.2°E) of the sampling area. Ephyrae relative abundance by station. Values in parenthesis indicate ephyrae abundance 100 m^-3^.

The preliminary analysis of the vertical distribution of *P*. *noctiluca* ephyrae revealed that practically all individuals (99.9%) were located in the upper 50 m of the water column (63.3% at 0–25 m and 36.6% at 25–50 m). The remaining 0.1% of ephyrae was found between 50 and 100 m, being absent below 100 m depth. Thus, the subsequent analyses, considering all the sampled stations, were focused on the first 50 metres. The GLMM analysis indicated that the light level (day/night) effect on ephyrae vertical distribution was significant (z = -3.85, p-value < 0.001) and that this effect of the light depended on depth (z = 3.09, p-value = 0.002; see S1 Table and S2 Fig). During the night, the ephyrae were mainly found in the upper 25 m, with low abundances at 25–50 m, whereas during the day they showed a more homogeneous distribution between surface and 50 m (Table 1; Fig 6). The vertical displacement of ephyrae during the day toward deeper water layers never crossed the lower limit of the thermocline and therefore, did not reach the DCM level (Fig 6).

**Table 1:** Ephyrae abundance (mean ± standard error) by day/night and depth stratum.

Ephyrae 100m^-3^	Night	Day
**0–25 m**	4.7 ± 2.90	7.6 ± 2.91
**25–50 m**	1.1 ± 0.91	6.7 ± 3.81

### Horizontal distribution of *P*. *noctiluca* ephyrae

**Fig 7:**
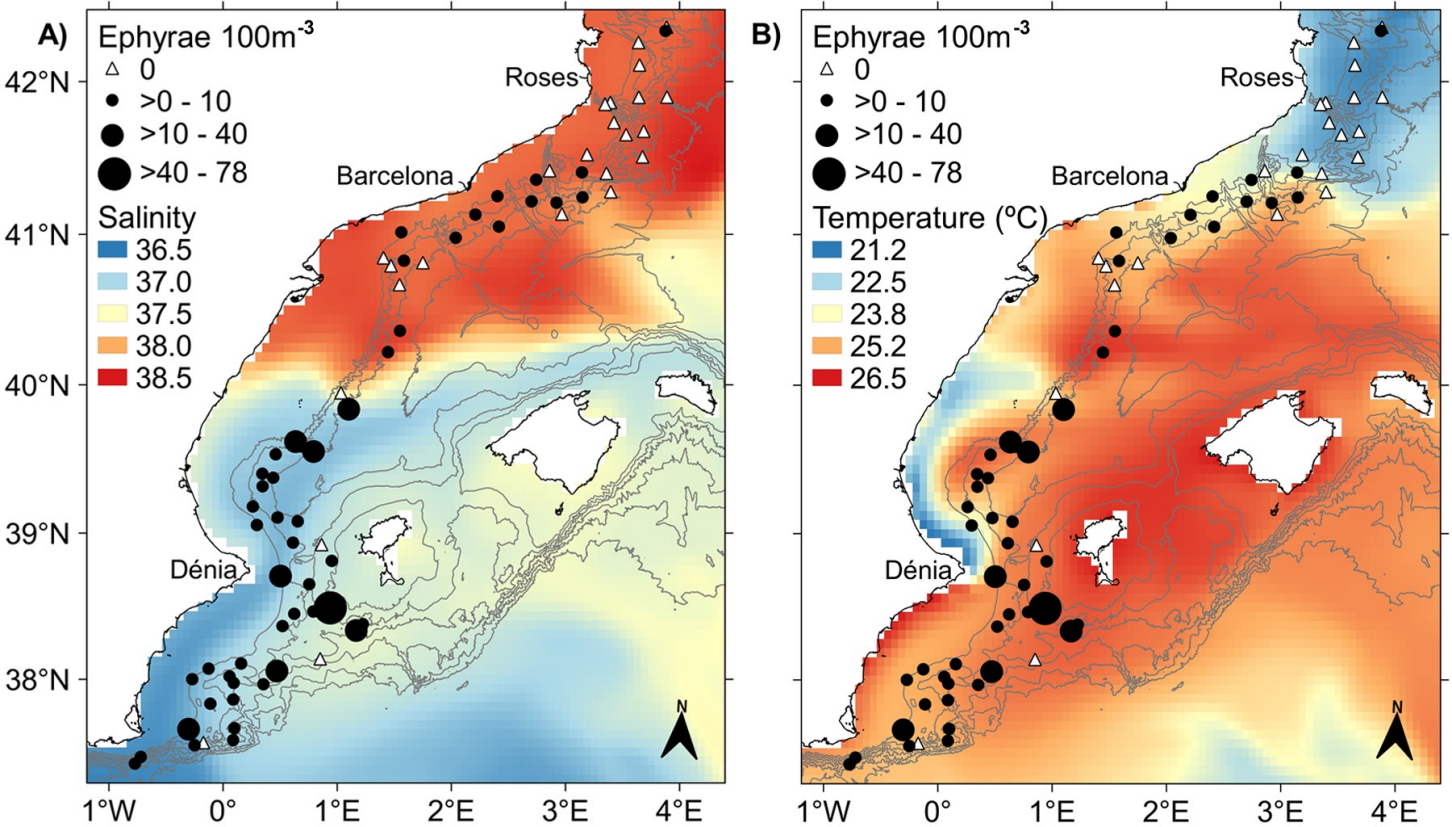
Horizontal distribution of *P*. *noctiluca* ephyrae overlaid on the mean a) salinity and b) temperature at 10 m depth for the sampling period from CMEMS reanalysis [45]. Coastline is from Instituto Hidrográfico de la Marina [52] and isobaths (every 400 m) are from EMODnet [43].

*P*. *noctiluca* ephyrae were particularly abundant south of 40.0°N, in coincidence with the presence of less saline surface water (Fig 7A). These high abundances also fit well with the path of the Balearic Current (Fig 5). In that area, ephyrae were present in almost all stations reaching the maximum abundance value (78 ephyrae 100 m^-3^ per station) slightly south of the Eivissa Channel (around 38.5°N; Fig 7). However, in the northern half of the area, occupied by the more saline waters, the abundance of ephyrae along the Northern Current path was much lower, being practically absent in the northernmost part characterized by the coldest temperatures (Fig 7B). This low abundance, between 40.0°N and 41.3°N, coincided with the mixture of waters from the Northern and Balearic Currents after the deflection of the Balearic Current at 4.0°E (Fig 5). The GLMM results showed that ephyrae abundance presented a negative association with salinity (z = -5.45, p-value < 0.001) and positive with temperature (z = 4.82, p-value < 0.001; see S2 Table and S3 Fig), with the highest values in the warm and low saline waters, between slightly south of the Eivissa Channel and 40.0°N (Fig 7).

**Fig 8:**
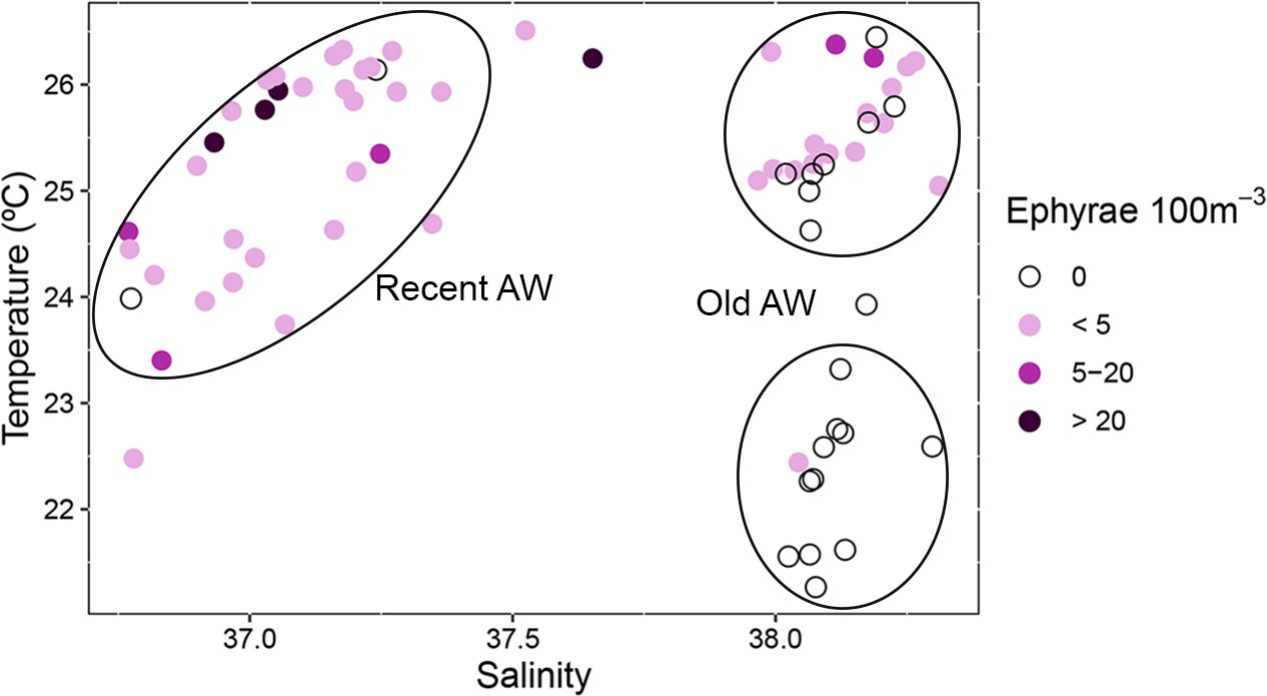
Depth-integrated ephyrae abundances in relation to mean surface (5–10 m depth) salinity and temperature at plankton stations.

Ephyrae abundance by station superimposed to surface salinity and temperature clearly defines three groups of stations (Fig 8). The highest abundance values were associated with recent AW, characterized by salinities lower than 37.5 and temperatures between 22.5°C and 26.5°C. Lower abundances were found associated to old AW (salinity >37.5) at temperatures between 24.0°C and 26.6°C, while ephyrae were practically absent in old AW with temperatures lower than 24.0°C (Fig 8). It is worth noting the high ephyrae abundance in a station not included in any of the three stations groups, located on the boundary between the low and high salinity waters, around 40.0°N (Fig 7A).

## Discussion

The results of the present study revealed a high variability in the spatial structure of *P*. *noctiluca* ephyrae in surface waters linked to the recent and old AW masses. The presence of *P*. *noctiluca* ephyrae in the upper water layer is in agreement with previous observations [28,53,54]. In the present study, however, we detected short-extent diel vertical migrations of ephyrae in these surface levels, with a wide distribution well above and within the thermocline during daytime, being more concentrated towards the surface at night. In any case, their vertical distribution was found far from the characteristic DCM that develops in the Mediterranean during the summer stratification period [55]. This vertical distribution contrasts with that described for the epipelagic zooplankton, characterized by the presence of a zooplankton maximum at approximately the same depths than the DCM during the daytime, whereas at night zooplankton ascends to upper layers [56–58]. This behaviour allows for feeding during the day at the DCM, avoiding the predation at the surface [59]. Taking into account that *P*. *noctiluca* ephyrae feed on a variety of zooplanktonic prey, such as copepoda, siphonophora, salpida or fish larvae [60,61], the migratory behaviour of the zooplankton would allow the overlap between *P*. *noctiluca* ephyrae and their prey during the night in surface waters. This would be supported by the high variety of prey detected in the gastric pouches of ephyrae during the night with respect to the day [62]. Moreover, we cannot discard the possibility that ephyrae might feed on microzooplankton, such as ciliates and flagellates, as observed in ephyrae of *Aurelia coerulea* [63]. These microzooplanktonic organisms are very abundant in the upper layers of stratified waters [64] and have been reported to be an important food source for other planktonic organisms, such as cladocerans and fish larvae, living in surface waters [65–67]. We can also consider that the vertical migration to slightly deeper and colder layers during the day would allow ephyrae to save energy, decreasing their metabolic rates and prey requirements [68,69]. The location of ephyrae in the illuminated levels of the water column could make them particularly vulnerable to visual predators such as crustaceans and fish [70,71]. However, the morphology and transparency of their bodies would make them barely visible to their predators [72]. All these strategies would allow *P*. *noctiluca* ephyrae to survive in this oligotrophic environment, avoiding the energy expense to cross the thermocline and reach the DCM zone where, furthermore, they would have to compete with other zooplankton species. The observed vertical migration of ephyrae would probably be constrained by the conditions of water column stratification. As vertical migration in jellyfish is accomplished by swimming [13], the weak swimming capability of ephyrae, in comparison with adult stages [73], may hinder large amplitude migration across the thermocline. *P*. *noctiluca* adults are strong swimmers that perform extensive vertical migrations from deep waters, below 300 m, during the day to the surface at night [34,74]. Although this migration can be attributed to feeding, following its zooplankton prey, other factors, such as the reproductive behaviour, could determine this migration pattern [34].

The horizontal distribution of *P*. *noctiluca* ephyrae showed a clear latitudinal gradient with high abundances in the southern part of the area, associated with the low saline recent AW. In summer, the surface AW which enters the Mediterranean may reach the Balearic Islands progressing northward across the Eivissa and Mallorca Channels [16,17] forming the warm fresh core of the Balearic Current. The ephyrae found in the recent AW along the Balearic Current path would have been generated by adults of *P*. *noctiluca* associated with the entrance of waters from the Atlantic into the Mediterranean, or trapped by the AW flow along its path. The maximum total body diameter of the collected ephyrae was 3.55 mm. According to Ramondenc *et al*. [75] their estimated age would be around 18 days, enough time to come from distant areas. However, we must consider that the instabilities of the current along the continental slope, that generate meanders and eddies [76], will ultimately determine the transport of ephyrae. Further modeling studies implementing individual-based models with a Lagrangian particle-tracking framework using hydrodynamic model outputs, will help to understand the trajectories and potential origin of these ephyrae. The highest abundance of ephyrae was detected in the warm recent AW at the Eivissa Channel, where the currents could have caused a funnel effect. As the recent AW inflow through Eivissa or Mallorca Channel presents not only seasonal [17] but also interannual variability [16], changes in the abundance of the species over time in the study area could be related to the interannual variability of this mechanism. We should consider that other hydrodynamic structures eastwards of the continental slope could also influence the ephyrae observed pattern, but the sampling strategy does not allow to test the possible effect of these processes. The transport of gelatinous organisms through the Strait of Gibraltar linked to the inflow of the AW into the Mediterranean has been previously reported for other species, such as *Rhizostoma luteum* [77]. The inflowing Atlantic jet involves high mesoscale activity [78], generating structures such as fronts and eddies that support high biological productivity [79–81]. These physical structures would favour gelatinous zooplankton advection and aggregation since, as osmoconformers that adjust to small salinity gradients, they tend to aggregate at density discontinuities [7–9,82].

In the northern half of the area, occupied by the old AW, the abundance of *P*. *noctiluca* ephyrae along the Northern Current path was much lower, being absent in the northernmost part. The low abundance of ephyrae contrasts with previous observations where high concentrations of *P*. *noctiluca*, adults and ephyrae, and other gelatinous organisms have been found associated to the Northern Current [9,28,34,35,83] in relation with the high levels of biological production in that area [36–38]. This scarcity of ephyrae together with their absence at the northern end of the area would suggest that the ephyrae found in the Northern Current would not come from areas further north but their presence would be related to the influx of the Balearic Current. The Balearic Current flowing to the northeast, carrying ephyrae, showed a deflection to the west around 4.0°E and joined the Northern Current probably supplying ephyrae to this current, where the mixture of waters would dilute their abundance. The absence of ephyrae in the northernmost part, characterized by the lowest temperature and more homogeneous upper layer, could suggest that temperatures in that area might have been too low for the development of the species. Nevertheless, taking into account the wide range of temperatures *P*. *noctiluca* tolerates [25,26] the temperature “per se” would not explain the absence of ephyrae. Previous works already reported the absence of ephyrae in that area in coincidence with high concentrations of anchovy larvae [35,84]. Sabatés *et al*. [35] argued that these colder waters come from further north, advected by the Northern Current, and involved a significant amount of waters from the shelf of the Gulf of Lions, an important spawning anchovy area [85]. Thus given the oceanic habitat of *P*. *noctiluca* [26], the origin of these waters would be the most likely explanation for the absence of ephyrae in that area.

Licandro *et al*. [31] already suggested that the seasonal occurrence of high densities of *P*. *noctiluca* adult swarms in the WM followed the progression of the AW surface stream, through the Strait of Gibraltar, along the North African coast before circulating anticlockwise around the WM basin [86]. In the present study, the possible flux of *P*. *noctiluca* ephyrae towards the north would depend on the seasonality of the currents pattern and on the high mesoscale variability of hydrodynamic processes [14,17]. The entrance of individuals through the Strait of Gibraltar would be supported by the similar genetic structure between *P*. *noctiluca* from the North Atlantic and the Mediterranean, resulting from an extensive gene flow and a high degree of connectivity between both populations [87,88]. Following the main currents in the WM, and considering the lifespan of this species, between 9 months and 1 year [27,28], individuals entering through the Strait of Gibraltar, or their offsprings, could act as sink populations at different Mediterranean regions (e.g. northern Adriatic, Naples Bay, Tunis Bay and Villefranche Bay [89]).

The frequency of *P*. *noctiluca* adult blooms in the Mediterranean has increased significantly since the 1990s, particularly in the western basin [26,32,33]. This increase has been related with climatic (mild winters, high temperature, low rainfall and high atmospheric pressure) and anthropogenic factors (lack of predators and decrease of pelagic fish populations, their competitors for food) [1,26,90]. However, we cannot rule out that these population increases could benefit from the contribution of individuals from the Atlantic across the Strait of Gibraltar. The results of the present study suggest the population connectivity of *P*. *noctiluca* between the Atlantic and the Mediterranean, a key issue for the understanding of the species population dynamics and its increasing abundance in the Mediterranean. Further observational and numerical simulation studies may contribute to better understand this potential connectivity and its seasonal and interannual variability.

## Supporting information

S1 Fig Measurement of total body diameter taken in *Pelagia noctiluca* ephyrae. (TIF)

S2 Fig Pearson residuals of GLMM analysis on the effect of light (day/night) and depth on the vertical distribution of *P. noctiluca* ephyrae. a) residuals distribution *vs*. fitted values; b) residuals spatial distribution (missing points in the north correspond to stations where ephyrae were absent); c) residuals distribution *vs*. light (D = day, N = night); d) residuals distribution *vs*. depth (m).
(PDF)

S3 Fig Pearson residuals of GLMM analysis on the effect of surface temperature and salinity on the horizontal distribution of *P*. *noctiluca* ephyrae. a) residuals distribution *vs*. fitted values; b) residuals spatial distribution; c) residuals distribution *vs*. temperature (°C); d) residuals distribution *vs*. salinity.
(PDF)

S1 Table Coefficients of GLMM analysis on the effect of light (day/night) and depth on the vertical distribution of *P*. *noctiluca* ephyrae. n.s. = non significant (p > 0.05).
(DOCX)

S2 Table Coefficients of GLMM analysis on the effect of surface temperature and salinity on the horizontal distribution of *P*. *noctiluca* ephyrae. (DOCX)

S1 Equation Mathematical model formulation for the GLMM analysis on the effect of light (day/night) and depth on the vertical distribution of *P*. *noctiluca* ephyrae (source [50]). (DOCX)

S2 Equation Mathematical model formulation for the GLMM analysis on the effect of surface temperature and salinity on the horizontal distribution of *P*. *noctiluca* ephyrae (source [50]). (DOCX)
