# Supplementary figures and images for: Spatial heterogeneity of Pelagia noctiluca ephyrae linked to water masses in the Western Mediterranean
Source: PLoS One. 2021 Apr 7;16(4):e0249756. doi: 10.1371/journal.pone.0249756 (PMC8026071; doi:10.1371/journal.pone.0249756)

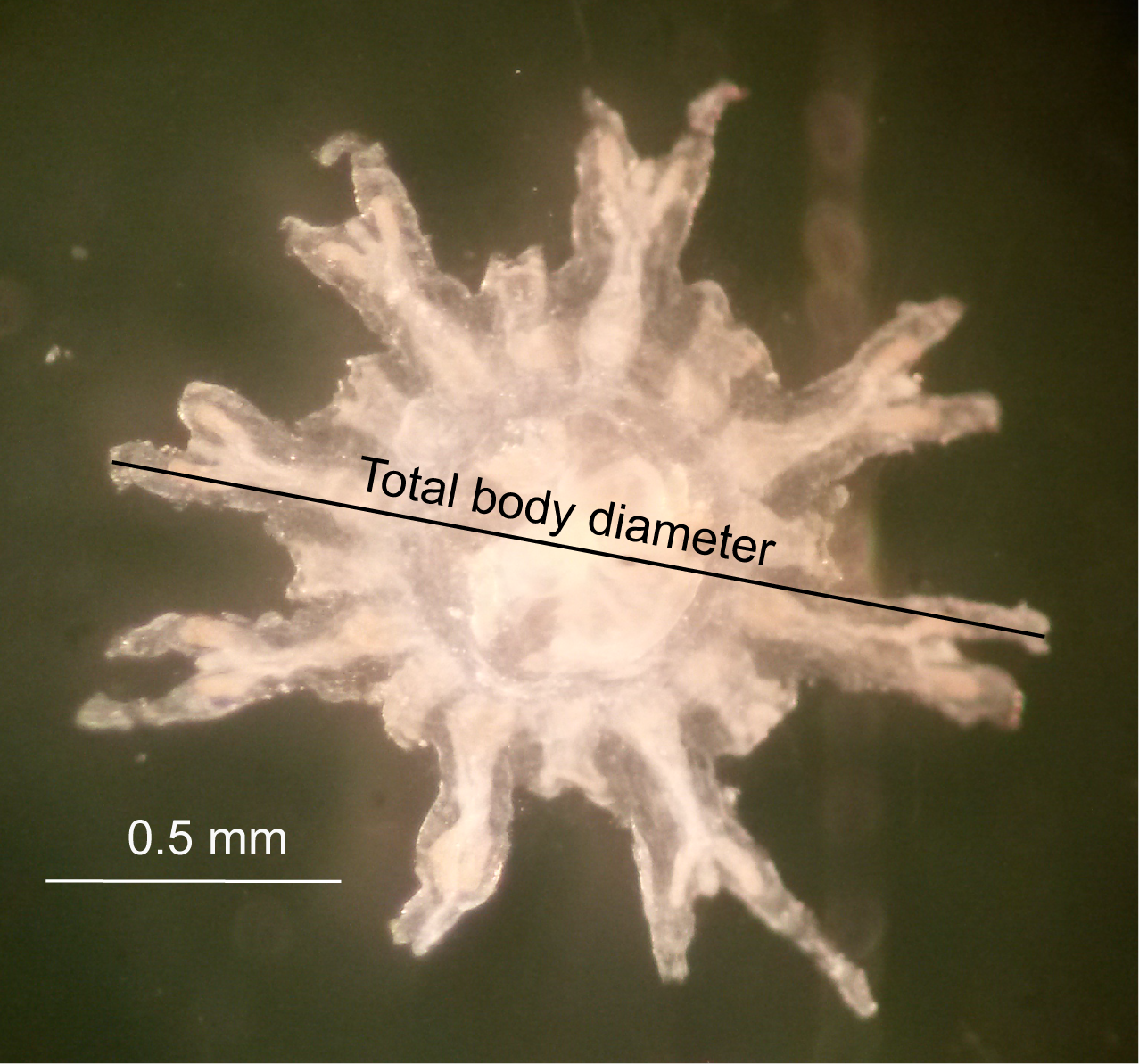

Supplement: S1 Fig — (TIF) [file pone.0249756.s001.tif]

A)

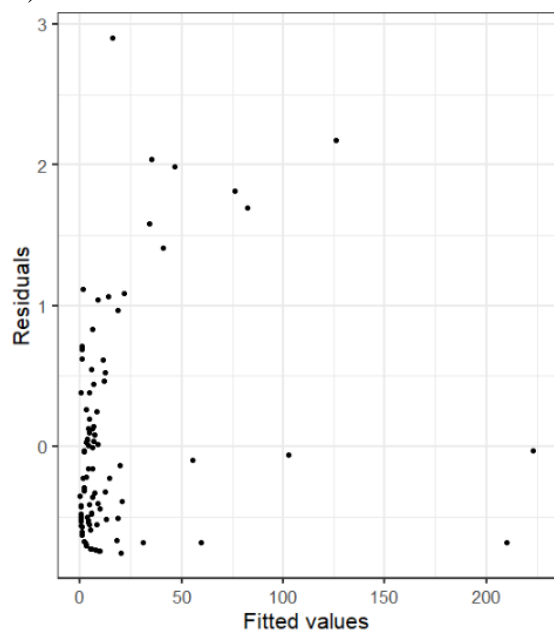

B)

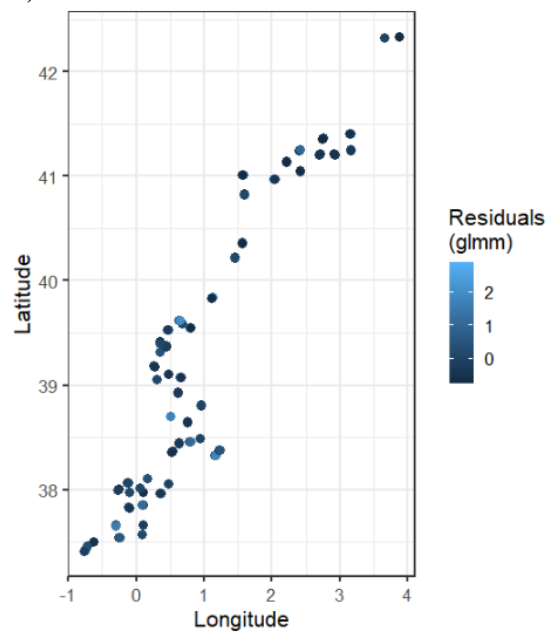

C)

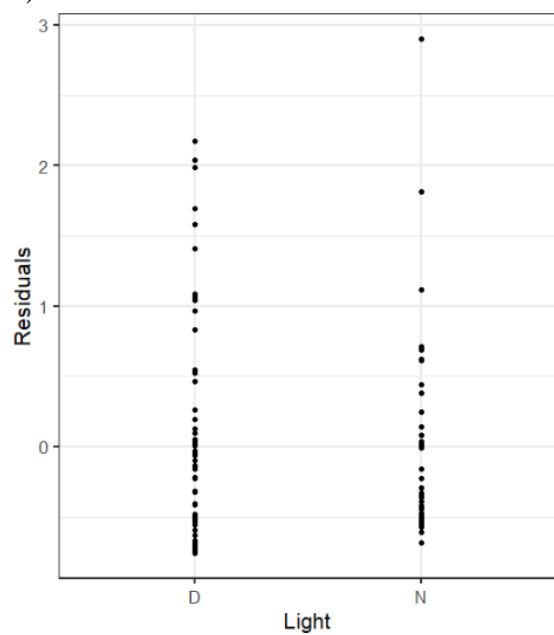

D)

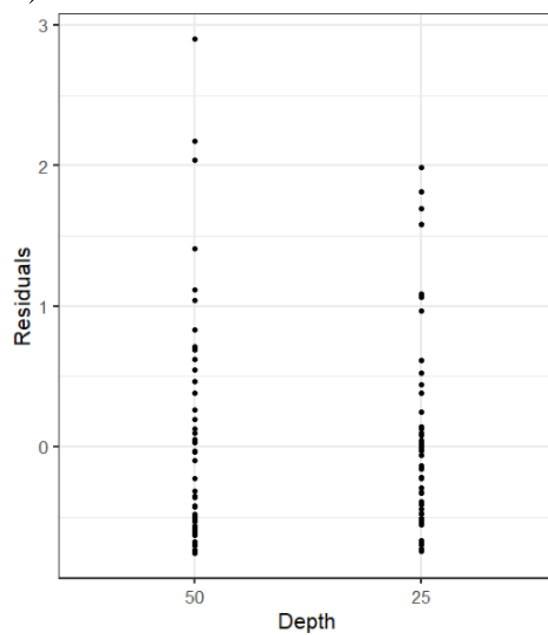

Supplement: S2 Fig — a) residuals distribution vs. fitted values; b) residuals spatial distribution (missing points in the north correspond to stations where ephyrae were absent); c) residuals distribution vs. light (D = day, N = night); d) residuals distribution vs. depth (m). (PDF) [file pone.0249756.s002.pdf]

A)

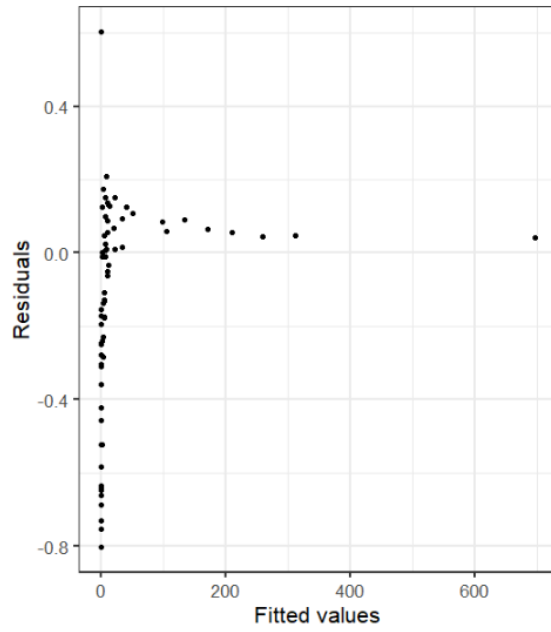

B)

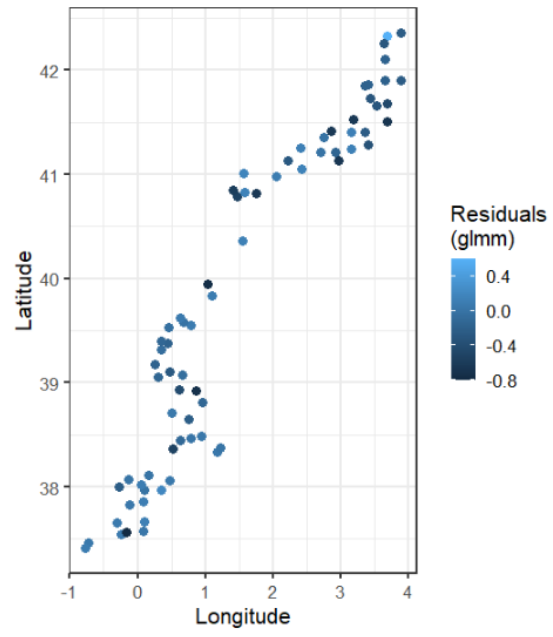

C)

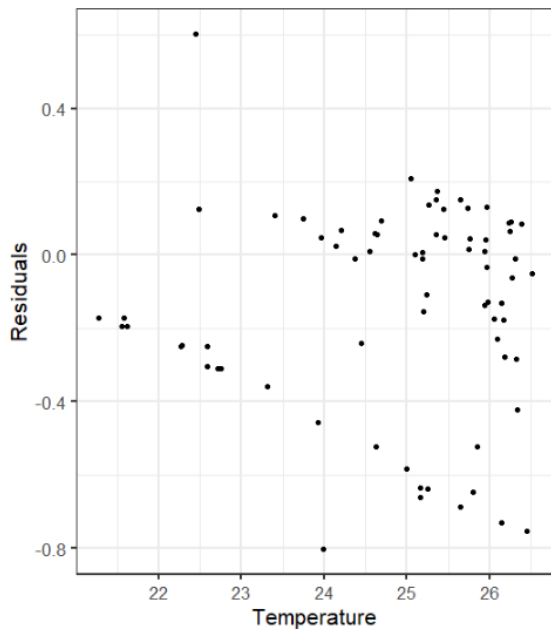

D)

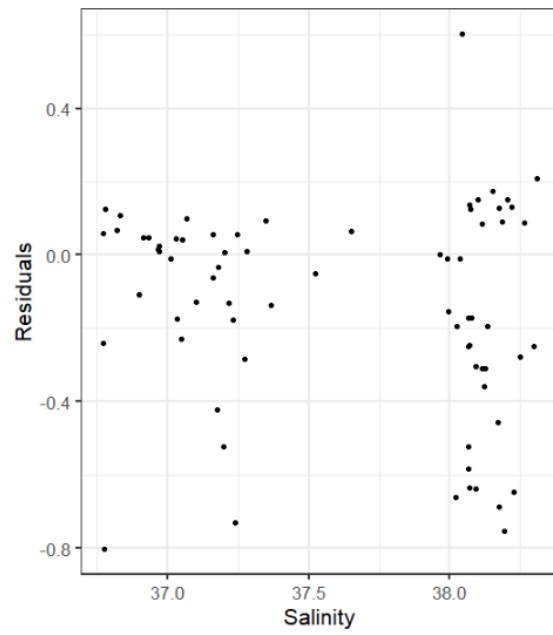

Supplement: S3 Fig — a) residuals distribution vs. fitted values; b) residuals spatial distribution; c) residuals distribution vs. temperature (°C); d) residuals distribution vs. salinity. (PDF) [file pone.0249756.s003.pdf]
