# Supplementary material for: Spatial heterogeneity of Pelagia noctiluca ephyrae linked to water masses in the Western Mediterranean
Source: PLoS One. 2021 Apr 7;16(4):e0249756. doi: 10.1371/journal.pone.0249756 (PMC8026071; doi:10.1371/journal.pone.0249756)
Supplement: S1 Table — n.s. = non significant (p > 0.05). (DOCX) [file pone.0249756.s004.docx]

|  | **Estimate** | **Std. Error** | **z value** | **p-value** |
| --- | --- | --- | --- | --- |
| **Intercept** | -4.32 | 0.03 | -142.29 | < 0.001 |
| **Light** | -2.04 | 0.53 | -3.85 | < 0.001 |
| **Depth** | 0.47 | 0.36 | 1.30 | 0.193 n.s. |
| **Light*Depth** | 2.07 | 0.67 | 3.09 | 0.002 |
