# Supplementary material for: Spatial heterogeneity of Pelagia noctiluca ephyrae linked to water masses in the Western Mediterranean
Source: PLoS One. 2021 Apr 7;16(4):e0249756. doi: 10.1371/journal.pone.0249756 (PMC8026071; doi:10.1371/journal.pone.0249756)
Supplement: S2 Table — (DOCX) [file pone.0249756.s005.docx]

|  | **Estimate** | **Std. Error** | **z value** | **p-value** |
| --- | --- | --- | --- | --- |
| **Intercept** | 43.57 | 13.69 | 3.18 | 0.001 |
| **Temperature** | 1.07 | 0.22 | 4.82 | < 0.001 |
| **Salinity** | -2.02 | 0.37 | -5.45 | < 0.001 |
