# Supplementary material for: Spatial heterogeneity of Pelagia noctiluca ephyrae linked to water masses in the Western Mediterranean
Source: PLoS One. 2021 Apr 7;16(4):e0249756. doi: 10.1371/journal.pone.0249756 (PMC8026071; doi:10.1371/journal.pone.0249756)
Supplement: Supplementary file 4 [file pone.0249756.s006.docx]

${NEphyrae}_{ij} \sim NB (μ_{ij},k)$ $E({NEphyrae}_{ij})=μ_{ij} and var({NEphyrae}_{ij}) =μ_{ij}+\frac{μ_{ij}^{2}}{k}$ $Log(μ_{ij}) ={Light}_{ij}+{Depth}_{ij}+ {Light}_{ij}×{Depth}_{ij}+offset+{Station}_{ij}$

${Station}_{ij} \sim N(0, σ^{2})$
